# Supplementary material for: Histone H3.3 promotes IgV gene diversification by enhancing formation of AID‐accessible single‐stranded DNA
Source: EMBO J. 2016 May 24;35(13):1452–64. doi: 10.15252/embj.201693958 (PMC4883027; doi:10.15252/embj.201693958)
Supplement: Supplementary file 1 — Expanded View Figures PDF [file EMBJ-35-1452-s001.pdf]

Expanded View Figures

WT [n=282]

		TO				
	%	T	C	G	A	Total
FROM	T		1	0	0	1
	C	11		19	1	**31**
	G	3	12		14	**29**
	A	0	0	0		0

h3.3 [n=364]

		TO				
	%	T	C	G	A	Total
FROM	T		0	0	0	0
	C	3		7	2	**12**
	G	3	4		2	**9**
	A	0	0	0		0

WT:UGI [n=155]

		TO				
	%	T	C	G	A	Total
FROM	T		3	0	0	3
	C	47		1	1	**49**
	G	0	2		40	**42**
	A	0	0	0		0

h3.3:UGI [n=126]

		TO				
	%	T	C	G	A	Total
FROM	T		0	0	0	0
	C	15		1	0	**16**
	G	0	0		12	**12**
	A	0	0	0		0

h3.3:H3.3 [n=92]

		TO				
	%	T	C	G	A	Total
FROM	T		0	0	0	0
	C	4		4	0	**8**
	G	2	3		5	**10**
	A	0	0	2		0

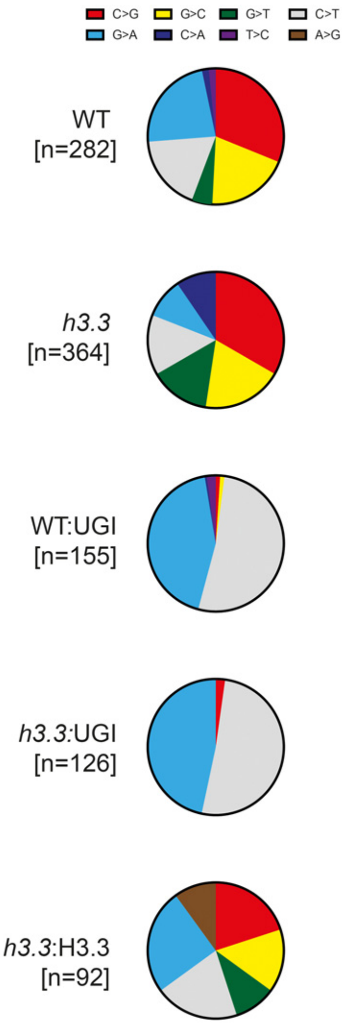

Figure EV1. The pattern of Ig point mutation is not affected by loss of H3.3.
Tables showing the pattern of non-templated point mutations in each cell line presented as absolute numbers in the table and as a proportion in the adjacent colour-coded pie chart. *n* gives the number of sequencing reads analysed.

 © 2016 MRC Laboratory of Molecular Biology

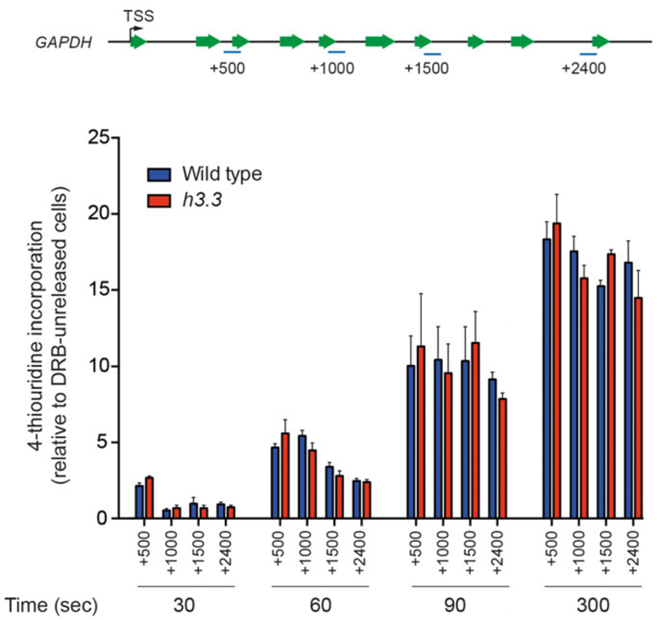

Figure EV2. Kinetics of transcription along the *GAPDH* locus measured by 4sUDRB.

4sUDRB mapping of transcription kinetics through the *GAPDH* locus. Representative locations of qPCR amplicons (blue) are shown relative to the *GAPDH* gene, and their distance from the TSS shown in parenthesis. qPCR signals are normalised to the enrichment at time zero (before release). Error bars represent ± SD of qPCR triplicates.

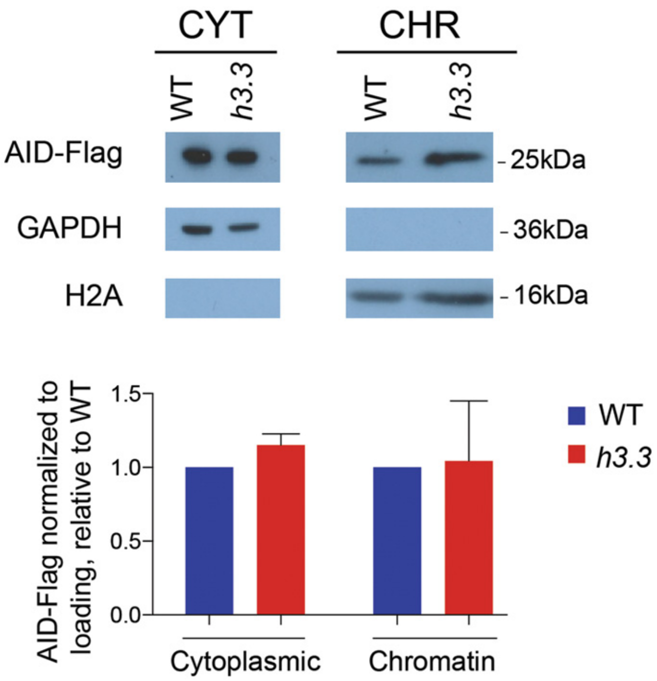

Figure EV3. The subcellular distribution of AID is not influenced by H3.3.

The subcellular localisation of AID is not grossly altered in the absence of H3.3. Detection of hAID^{up} in cytoplasmic and chromatin fractions of WT and *h3.3* cells that overexpress hAID^{up} to around 30 times the WT AID level. GAPDH is used as a cytoplasm-specific control and H2A as a chromatin-specific control. The graphs show the quantification of two biological repeats with ± SD.

© 2016 MRC Laboratory of Molecular Biology

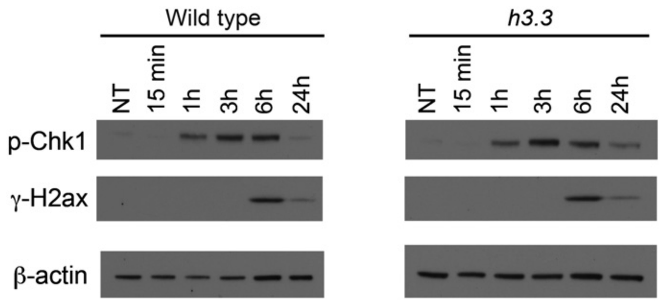

Figure EV4. The kinetics of CHK1 and H2Ax phosphorylation following exposure to UV light is not altered by the absence of H3.3.
Phospho-Chk1(S345) and γH2Ax, detected by Western blot in total protein extract of WT and *h3.3* cells, at the indicated time points post-UVC irradiation (265 nm at 8 J/m^2). β-actin is shown as a loading control.

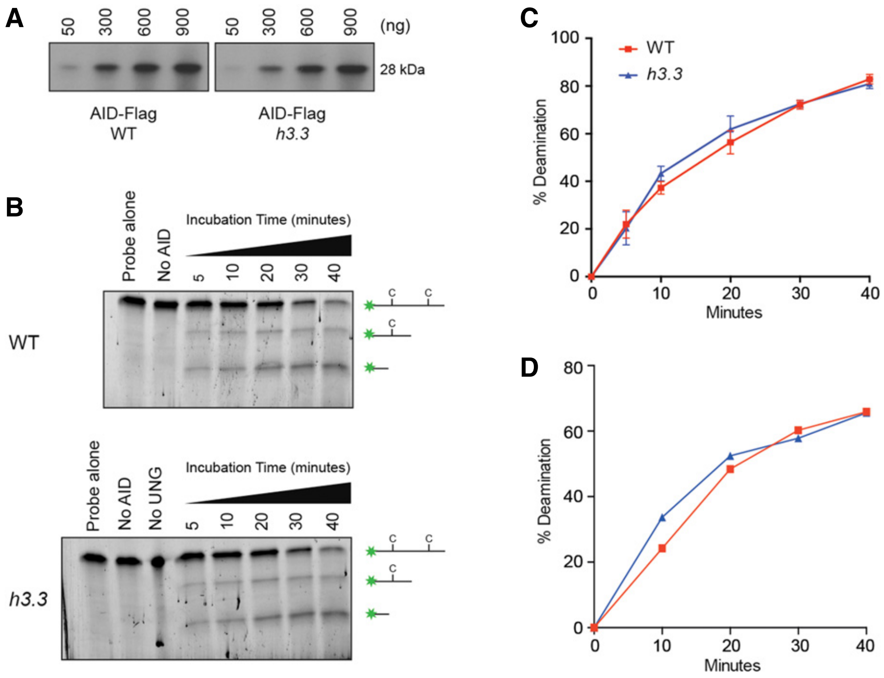

Figure EV5. hAID^{up} protein pulled down from wild-type and *h3.3* cells is equally active.

- A Western blot confirmation of colorimetric quantification of hAID^{up} protein pulled down from wild-type and *h3.3* cells.
- B 10% TBE-urea gel image of *in vitro* deamination assay with hAID^{up} protein pulled down from wild-type and *h3.3* cells. About 600 ng of hAID^{up} was incubated with fluorescein-labelled oligonucleotides containing two dC bases. Treatment with UNG renders deaminated sites sensitive to cleavage in alkali and the resulting fragments are visualised on the gel.
- C Quantification of the deamination assay shown as a Michaelis–Menten graph of two independent experiments. Error bars represent ± SD of two independent experiments.
- D Quantification of the deamination assay done with hAID^{up} pulled down from the total nuclear fraction, shown as a Michaelis–Menten analysis of a single experiment.

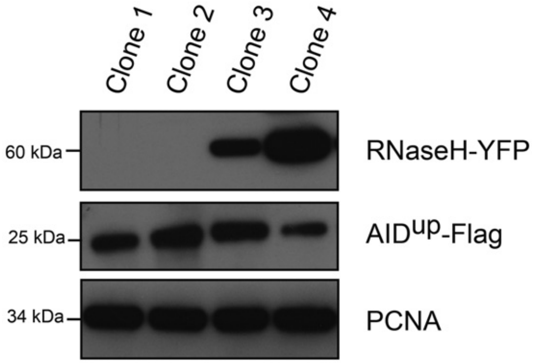

Figure EV6. Expression of RNase HI in DT40 cells.
Western blot for expression of hAID^{up}-FLAG (anti-FLAG) and chicken RNase HI-YFP (anti-YFP) in the four DT40 clones studied in Fig 5C. PCNA provides a loading control.

 © 2016 MRC Laboratory of Molecular Biology
