# Supplementary material for: Histone H3.3 promotes IgV gene diversification by enhancing formation of AID‐accessible single‐stranded DNA
Source: EMBO J. 2016 May 24;35(13):1452–64. doi: 10.15252/embj.201693958 (PMC4883027; doi:10.15252/embj.201693958)
Supplement: Supplementary file 2 — Table EV1 [file EMBJ-35-1452-s002.docx]

**Table EV1**

| **AID regulator** | **Function** | **Reference** |
| --- | --- | --- |
| Hsp90 | AID stabilization in the cytoplasm | Keim et al, 2013 |
| REGγ | AID degradation in the nucleus | Keim et al, 2013 |
| PTBP2 | Binding of AID to S regions | Keim et al, 2013 |
| 14-3-3 | Targeting and stabilsation of AID and CSR cofactors | Keim et al, 2013 |
| RPA | Targeting of phosphorylated AID to ssDNA | Keim et al, 2013 |
| Spt5 | Interaction with stalled RNA PolII | Keim et al, 2013 |
| RNApolII | Targeting of AID to transcribed substrates | Keim et al, 2013 |
| RNA exosome complex | Targeting of AID to both DNA strands | Keim et al, 2013 |
| PKA/PKC | Phosphorylation of AID at S38 and T140 | Sun et al, 2013 |
| mir-155/mir-181b/mir-93 | Interaction with 3’UTR of AID to block AID expression | Sun et al, 2013 |
| GANP | Modification of chromatin structure and enhancement of AID recruitment | Singh et al, 2013 |

**Table EV1. List of know AID regulators**. None of these known AID regulators show altered expression in *h3.3* compared to WT cells of more than 2-fold (p<0.001, negative binomial test). The complete RNA-seq data set (Frey et al, 2014) can be found in Array Express, with accession number EMTAB-2754.
