# Supplementary material for: Histone H3.3 promotes IgV gene diversification by enhancing formation of AID‐accessible single‐stranded DNA
Source: EMBO J. 2016 May 24;35(13):1452–64. doi: 10.15252/embj.201693958 (PMC4883027; doi:10.15252/embj.201693958)
Supplement: Supplementary file 3 — Table EV2 [file EMBJ-35-1452-s003.docx]

**Table EV2. List of oligonucleotides.**

Primers for ChIP, DRIP and expression qPCRs

| Oligo Name | Oligo sequence (5’ to 3’) | Reference |
| --- | --- | --- |
| IgV_rearranged_Fw | CCCTTCACGATTCTCCGGTT | This study |
| IgV_rearranged_Rev | GTCAGCGACTCACCTAGGAC | This study |
| IgV_unrearranged_Fw | CGAGGCTGTCTATTACTGTGGG | This study |
| IgV_unrearranged_Rev | GGAATGGAGGTGGGACTTGT | This study |
| ρ-globin_exon_Fw | GACAATGCAAGGATGGAGTGG | Wang et al,2005 |
| ρ-globin_exon_Rev | GAGATGCTCTTTCTGCAGGAAAAT | Wang et al, 2005 |
| GAPDH_H3.3ctrl_Fw | AGCCAAAAGGTGTGTTGGAG | This study |
| GAPDH_H3.3ctrl_Rev | GCACACACCCATCAGACAAG | This study |
| AID_Expression_Fw | GGACAGCCTCTTGATGA | This study |
| AID_Expression_Rev | GATGTAGCACTGTCACG | This study |
| UGI_Expression_Fw | TGGGAATAAACCAGAAAGTGA | This study |
| UGI_Expression_Rev | AGCCCAAGGTTTATATTCTGGAG | This study |
| EF1α_Expression_Fw | GGTTATGCCCCTGTGCTGGATT | This study |
| EF1α_Expression_Rev | CTTCTTGTCGACGGCCTTGATGA | This study |

Primers for mutation analysis

| Oligo Name | Oligo sequence (5’ to 3’) | Reference |
| --- | --- | --- |
| JSCVLF5 | ATTGGATCCGGCTCTGTCCCATTGCTGCGCGG | Sale et al, 2001 |
| JSCVLR3 | ATTGAATTCCCCCAGCCTGCCGCCAAGTCCAAG | Sale et al, 2001 |
| AID_Sequencing_Fw | GTTTCTTGAATTCAGCTTGAATTCGCCACCATGGAC | This study |
| AID_Sequencing_Rev | TCCAAGCGGCTTCGGCCAGTAACG | This study |
| Bcl6_Sequencing_Fw | ATTGGATCCGCAGCCTCGTCTCTATCTATAGA | This study |
| Bcl6_Sequencing_Rev | ATTGAATTCGATGCCTACAAAGTATCAGGAGG | This study |
| EF1α_Sequencing_Fw | ATTGGATCCGGCTATACAGAGGGCTACAATCGAC | This study |
| EF1α_Sequencing_Rev | ATTGAATTCCGGTGATGCCATTCTCATACCTT | This study |

Primers for Nucleosome positioning assay qPCRs

| Oligo Name | Oligo sequence (5’ to 3’) | Reference |
| --- | --- | --- |
| MNase_24_Fw | GACACACAGCTGCTGGGATTCCGCCATGGC | This study |
| MNase_24_Rev | CACAGTCCCCGACCAGGCGCAACGAGTACC | This study |
| MNase_54_Fw | CTGGGCTCCTCTCCTCCTGGCGG | This study |
| MNase_54_Rev | CCGCGCAGCAATGGGACAGAGC | This study |
| MNase_84_Fw | CCACACCTCAGGTACTCGTTGC | This study |
| MNase_84_Rev | TCAGTGACGGCCCCGCACGCAC | This study |
| MNase_114_Fw | GGGGACTGTGGGCACGGGGCTCTGTCCCAT | This study |
| MNase_114_Rev | AGGGAGAGGAGAGAGGGGAGAAAACGGCAA | This study |
| MNase_178_Fw | TGCTGCGCGGGCAGGGCTGTGC | This study |
| MNase_178_Rev | TCAGCGCTGCCTGCACCAGGGA | This study |
| MNase_204_Fw | TTGCCGTTTTCTCCCCTCTC | This study |
| MNase_204_Rev | GGTTTGCTGACACCGAGGA | This study |
| MNase_238_Fw | TCCTCTCCCTCTCCAGGTTCCCTGGTGCAG | This study |
| MNase_238_Rev | CCCCGGAGCAGGTGATCTTGACGGTTTCTC | This study |
| MNase_294_Fw | GCAGCGCTGACTCAGCCGTCCTC | This study |
| MNase_294_Rev | CTTCTGCTGGTACCAGCCATAGT | This study |
| MNase_324_Fw | GGGAAAGTTATAGGAGCTACTAT | This study |
| MNase_324_Rev | GTTGGTGTTAGCATAGATCACAG | This study |
| MNase_351_Fw | CTAACACCAACAGACCCTCGG | This study |
| MNase_351_Rev | TTTGGAACCGGAGAATCGTGA | This study |
| MNase_385_Fw | GCTAACACCAACAGACCCTCGGACATCCCTTC | This study |
| MNase_385_Rev | GCCGACGACGAGGCTGTCTATTACTGTGGG | This study |
| MNase_413_Fw | TCCAAATCCGGCTCCACAG | This study |
| MNase_413_Rev | TGTCTGCACTCCCACAGTAA | This study |
| MNase_474_Fw | GAGGCTGTCTATTACTGTGGGAGTGCAGAC | This study |
| MNase_474_Rev | CGAGACGAGGTCAGCGACTCACCTAGGACG | This study |
| MNase_504_Fw | AGCAGCAGTACTGCTGCTTTGGGGCCGGGA | This study |
| MNase_504_Rev | GTCACAATTTCACGATGGGGGAAGAAAGAC | This study |

Primers for bisulphite mapping of ssDNA

| Oligo Name | Oligo sequence (5’ to 3’) | Reference |
| --- | --- | --- |
| Bisulfite_Map_Fw | ATTGGATCCGCACACCTCAGGTACTCGTTG | This study |
| Bisulfite_Map_Rev | ATTGAATTCGAAGAAAGACCGAGACGAGG | This study |

Oligo for *in-vitro* AID activity assay

| Oligo Name | Oligo sequence (5’ to 3’) | Reference |
| --- | --- | --- |
| AID_Activity | [Flc]ATATGAATAGAATAGAGGGGTGAGCTGGGGTGAGCTGGGGTGA | Wang et al, 2009 |

Primers for measurement of transcription elongation speed of RNA Polymerase II by 4sUDRB.

| Oligo Name | Oligo sequence (5’ to 3’) | Reference |
| --- | --- | --- |
| IgV_rearranged_Fw | CCCTTCACGATTCTCCGGTT | This study |
| IgV_rearranged_Rev | GTCAGCGACTCACCTAGGAC | This study |
| IgV_constant_Fw | TGGATTGGGTGATCGATGGC | This study |
| IgV_constant_Rev | TAGAGGTGCCGTTGTGTGTG | This study |
| IgV_TSS_Fw | CGGAAGGACGCGGGTATAAA | This study |
| IgV_TSS Rev | ACCAGGCGCAACGAGTAC | This study |
| IgV_Intron_Fw | TGGTCTCTCACTGGGGACTC | This study |
| IgV_Intron_Rev | GCACTTACCTGGACAGCTGA | This study |
| Gapdh_500_Fw | CACTGTCAAGGCTGAGAAC | This study |
| Gapdh_500_Rev | CTGGAGGGCATTTACTTTCATC | This study |
| Gapdh_1000_Fw | TGTTTGTGATGGGTGTCAAC | This study |
| Gapdh_1100_Rev | GCATTGCTGGGAAAGAAAGAAG | This study |
| Gapdh_1500_Fw | TGAATGGGTGAGTGTGGTC | This study |
| Gapdh_1500_Rev | CAGCAGGGAACAGCATAAG | This study |
| Gapdh_2400_Fw | CTGTGTTCAGGTGATTCCAG | This study |
| Gapdh_2400_Rev | AAAGAAAGTAGGCAGCATCC | This study |
